# Rho-associated kinase1 promotes laryngeal squamous cell carcinoma tumorigenesis and progression via the FAK signaling pathway

**DOI:** 10.1007/s12672-022-00561-7

**Published:** 2022-10-05

**Authors:** Liyun Yang, Peipei Qiao, Jianwei Zhang, Shuixian Huang, An Hu

**Affiliations:** Department of Otolaryngology, Gongli Hospital, The Second Military Medical University, Shanghai, 200135 China

**Keywords:** LSCC, ROCK1, Tumorigenesis and progression, FAK

## Abstract

Laryngeal squamous cell carcinoma (LSCC) is one of the most common head and neck squamous cell carcinomas (HNSCC). Rho-associated kinase1 (ROCK1) is considered to promote progression of numerous cancers, however, its role in LSCC is still unknown. Here, the expression level of ROCK1 is higher in LSCC tissues than non-tumor tissues, and the expression level of ROCK1 is positively correlated with advanced stage and poor survival prognosis. ROCK1 knockdown in TU686 and TU212 cells dramatically inhibits cellular proliferation, migration and invasion. Whereas the overexpression of ROCK1 reversed these changes. FAK signaling pathway plays an essential role in promoting LSCC progression. Inhibiting FAK activity with TAE226 observably impairs the tumor-promoting effects. In conclusion, ROCK1 promotes LSCC tumorigenesis and progression via the FAK signaling pathway, targeting the ROCK1 molecule may represent potential targets for clinical LSCC treatment.

## Introduction

Laryngeal squamous cell carcinoma (LSCC) is one of the most common head and neck squamous cell carcinomas (HNSCC) [1]. World has made great strides in LSCC treatment, nonetheless the 5-year survival rate of LSCC patients remains 68% [2]. Therefore, clarifying the mechanism underlying tumorigenesis and progression is of great significance for developing effective clinical prevention programs and new targeted therapies for LSCC.

Rho-associated kinase1 (ROCK1) is a classic serine/threonine protein kinase. ROCK1 can regulate downstream substrates such as muscle Globulin, adducin and protein kinases [3]. ROCK1 also can phosphorylate various substrates such as myosin light chain (MLC) phosphatase, LIM kinase, PTEN, insulin receptor substrate (IRS), ezrin/radixin/moesin (ERM) proteins, and JNK interacting protein (JIP-3) [4–6]. Recently, accumulating evidence has indicated that ROCK1 plays a crucial role in tumorigenesis and progression. Overexpression or activation of ROCK1 increases proliferation, while inhibition of ROCK1 by interfering RNA or inhibitors markedly decreases migration [7]. Stadler et al. also find that CAFs in the tumor microenvironment promote colorectal cancer metastasis via targeting ROCK1 [8]. Although ROCK1 makes a contribution to tumorigenesis and progression, its role in LSCC is still unknown. Therefore, we sought to investigate the expression and prognostic value of ROCK1 in LSCC patients, and to explore the role of ROCK1 in tumorigenicity.

In the present study, the expression level of ROCK1 is higher in LSCC tissues than non-tumor tissues, and the expression level of ROCK1 is positively correlated with advanced stage and poor survival prognosis. ROCK1 knockdown in TU686 and TU212 cells dramatically inhibits cellular proliferation, migration and invasion. Whereas ROCK1 over-expression reversed these changes. FAK signaling pathway plays an essential role in promoting LSCC progression. Inhibiting FAK activity with TAE226, which is a specific FAK inhibitor, observably impairs the tumor-promoting effects. Those findings indicate that ROCK1 promotes LSCC tumorigenesis and progression via the FAK signaling pathway, targeting the ROCK1 molecule may represent potential targets for clinical LSCC treatment.

## Materials and methods

### Patient samples

Laryngeal cancer tissues and matched adjacent non-cancer tissues samples of 30 patients were collected from the Gongli Hospital (the Second Military Medical University), Ruijin Hospital (Shanghai Jiao Tong University School of Medicine) and Shanghai Ninth People’s Hospital (Shanghai Jiaotong University School of Medicine) between 2019 and 2021. The tissue samples from those patients were confirmed by pathological diagnosis. The corresponding non-tumor location was at least 5 cm from the laryngeal tumor. Patients with critical organ exhaustion, severe hypertension, diabetes and mental disorders were excluded in our study.

### Immunohistochemical (IHC)

LSCC tissues samples of 30 patients were first fixed in formalin and embedded in paraffin, then they were xylene-dewaxed and treated in citrate buffer (0.01 mol/L, pH 6.0). Rabbit anti-ROCK1 antibody (1:150, Cell Signaling Technology) was used to stain those samples at 4 °C overnight. Then samples were incubated in secondary antibody (1:150, Cell Signaling Technology) for 30 min at 37 °C, and finally visualized with DAB solution and counterstained with haematoxylin. Two senior pathologists unaware of clinical information evaluated samples, and a third pathologist was invited to reevaluate the disputed samples. Five typical viewpoints were selected for IHC observation under a light microscope. The score of positive cells was classified by 4 grades (%): grade 0 (< 10%), grade 1 (10–30%), grade 2 (30–50%), grade 3 (> 50%). The score of staining intensity was also divided into 4 grades (intensity): grade 0 (no staining), grade 1 (weak staining), grade 2 (moderate staining) and grade 3 (strong staining). Total immunohistochemical score = positive cell score + staining intensity score. The total scores 0, 1–2, 3–4, and 5–6 were defined as negative (−), weak positive (±), moderate positive (+), and strong positive (++) respectively.

### Cell lines

Cells were preserved by the Shanghai Institutes for Biological Sciences, Chinese Academy of Sciences. Dulbecco’s Modified Eagle’s Medium (DMEM, Gibco company, USA) with 10% Fetal Bovine Serum (FBS, Gibco) were used to culture the above cells. And cells were cultured in a humidified cell incubator with an atmosphere of 5% CO_2_ at 37 °C.

### Immunofluorescent (IF)

5 × 10^4^ cells were seeded into slides (Millipore, MA, USA) and fixed with 4% paraformaldehyde (PFA) for 30 min. PBS was used to rinse the slides for 3 times, and 5% BSA was applied to block the slides for 1 h at 37℃ and primary antibodies were used to incubate the slides at 4 °C overnight. Next day, slides were rinsed with PBS for 3 times and incubated with secondary antibodies in the dark at 37℃ for 1 h. Primary antibody included Anti-ROCK1 (Cell Signaling Technology) and Alexa Fluor® 488 goat (Abcam, Cambridge, MA, USA) and secondary antibody included Alexa Fluor® 555 goat anti-rabbit IgG. DAPI was used to visualize the nuclei of cells in the dark for 5 min. Analyzing slides by fluorescent microscopy (10x).

### Transfections

3 × 10^5^ cells were seeded into 6-well plates and incubated for all night. ROCK1 plasmid (Myc-ROCK1-Delta3 (1-727), Gene Pharma Company, Shanghai) by lipofectamine 2000 (Invitrogen) was used to transfect, and then transfected cells were selected via 1200 ug/ml G418. Selected clones were verified by Western Blot and frozen.

### RNA extraction and quantitative real-time PCR (qRT-PCR)

Total RNA was extracted using Trizol reagent (Invitrogen, Carlsbad, CA, USA) and cDNA was synthesized using the Reverse Transcription kit (Promega, Madison, WI, USA). qRT-PCR was performed to quantify ROCK1 mRNA level with the SYBR Green PCR core Reagent kit (Applied Biosystems, Foster city, CA, USA). GAPDH was used as the endogenous reference. Data were analyzed by using the comparative Ct method. Specificity of resulting PCR products was confirmed by melting curves. The primers were designed using Primer Express v2.0 software (Applied Biosystems, Foster City, CA, USA). The primers used in this assay were: ROCK1 forward 5′-AGG AAG GCG GAC ATA TTA GTC CCT-3′ and reverse 5′-AGA CGA TAG TTG GGT CCC GGC-3′, β-actin forward 5′-TGA CGT GGA CAT CCG CAA AG-3′ and reverse 5′-CTG GAA GGT GGA CAG CGA GG-3′.

### Western blot analysis

Total proteins were lysed with RIPA buffer (Pierce, Rockford, USA). And BCA Protein Assay Kit was performed to measure the concentration of protein. Proteins with an equal amount (100 µg/sample) were electrophoresed by 10% SDS-PAGE for 2 h and transferred onto 0.22 μm PVFD membranes (Millipore, MA, USA). Incubating membranes with primary antibodies for all night at 4℃ which included anti-ROCK1 (1:2000, Cell Signaling Technology), anti-p-FAK (1:2000, Cell Signaling Technology), anti-FAK (1:2000, Cell Signaling Technology) and GAPDH (1:5000, Abcam). Then incubating membranes with secondary antibody (1:5000, Cell Signaling Technology) at 4℃ for 2 h, the proteins were visualized using enhanced chemiluminescence detection system (Amersham Bioscience, Piscataway, NJ, USA).

### Plate colony formation assay

Seeding cells into 6-well plates at 1 × 10^3^ and 2 × 10^3^ cells/well. Cells were cultured in DMEM with 10% FBS for 3 weeks, washed twice with PBS and stained with crystal violet for 30 min. Cell colonies were counted in every well.

### Apoptosis levels using annexin V/PI staining

TU686 and TU212 cells, with different treatments, were treated with celastrol (1, 2 and 4 µM) for 48 h, and harvested and washed with PBS for 3 times. Then cells were resuspended with 1X binding buffer prior for half an hour at 37 °C in the dark. Cells were then double-stained with PI for 30 min at 37 °C in the dark. The apoptotic cells were quantitatively counted by a flow cytometer.

### In vitro cell migration and invasion assays

2 × 10^5^ TU686 and TU212 cells, with different treatments, were seeded in the 200 µl of serum-free DMEM. And 600 µl DMEM of containing 10% FBS was added to the lower chamber. The insert chambers’ membrane was coated by Diluted Matrigel (BD Biosciences) for measuring the cells invasion. TU686 and TU212 cells, with different treatments, were counted under a high-power objective (10x) in random fields.

### Animal experiments

Animals were obtained from the Institute of Zoology, Chinese Academy of Sciences. Animal experiments were carried out in accordance with the Guidelines for “The Care and Utilization of Experimental Animals”. The following protocols had been approved by the medical center IRB. Animal care and treatment were conducted in accordance with NIH guidelines for “The Care and Utilization of Laboratory Animals”. At the end of the experiment, an overdose of sodium pentobarbital (4%, 200 mg/kg; Sigma, Shanghai, China) was performed to kill the animals by intraperitoneal injection. And lung specimens were collected from each group for further analysis.

### In vivo metastasis

A 4-week-old male immunodeficient mouse reared at the Animal Resource Facility of Shanghai Jiao Tong University School of Medicine. The animal care and experiment were carried out in accordance with the Guidelines for “The Care and Utilization of Experimental Animals” and “The Principles for the Care and Utilization of Vertebrates”, and approved by the Experimental Animal Ethics Committee of Shanghai Jiao Tong University School of Medicine. Experimental animals were grouped according to the randomization formula. In the allocation process, researchers did not understand the experimental stages, the experimental progress, the results evaluation, and the data analysis. The mice were randomly divided into groups (5 in each group): TU686 and TU212 cells with different treatments were injected via tail vein into the mice. After 6 weeks, H&E staining was performed to count pulmonary metastatic nodules. For IH staining, the target recovery solution was used to thermally induce epitope recovery in microwave for 6 min. At high magnification (×100), the number of tumors was calculated in the field.

### Statistical analysis

Data were analyzed by Graph-Pad Prism 6 software and presented by means ± SD. Student’s t test and one-way analysis of variance (ANOVA) were used to analyze the data and the significance level was set at P < 0.05.

## Results

### ROCK1 is overexpressed in LSCC tissues and cells and related to poor survival

**Fig. 1:**
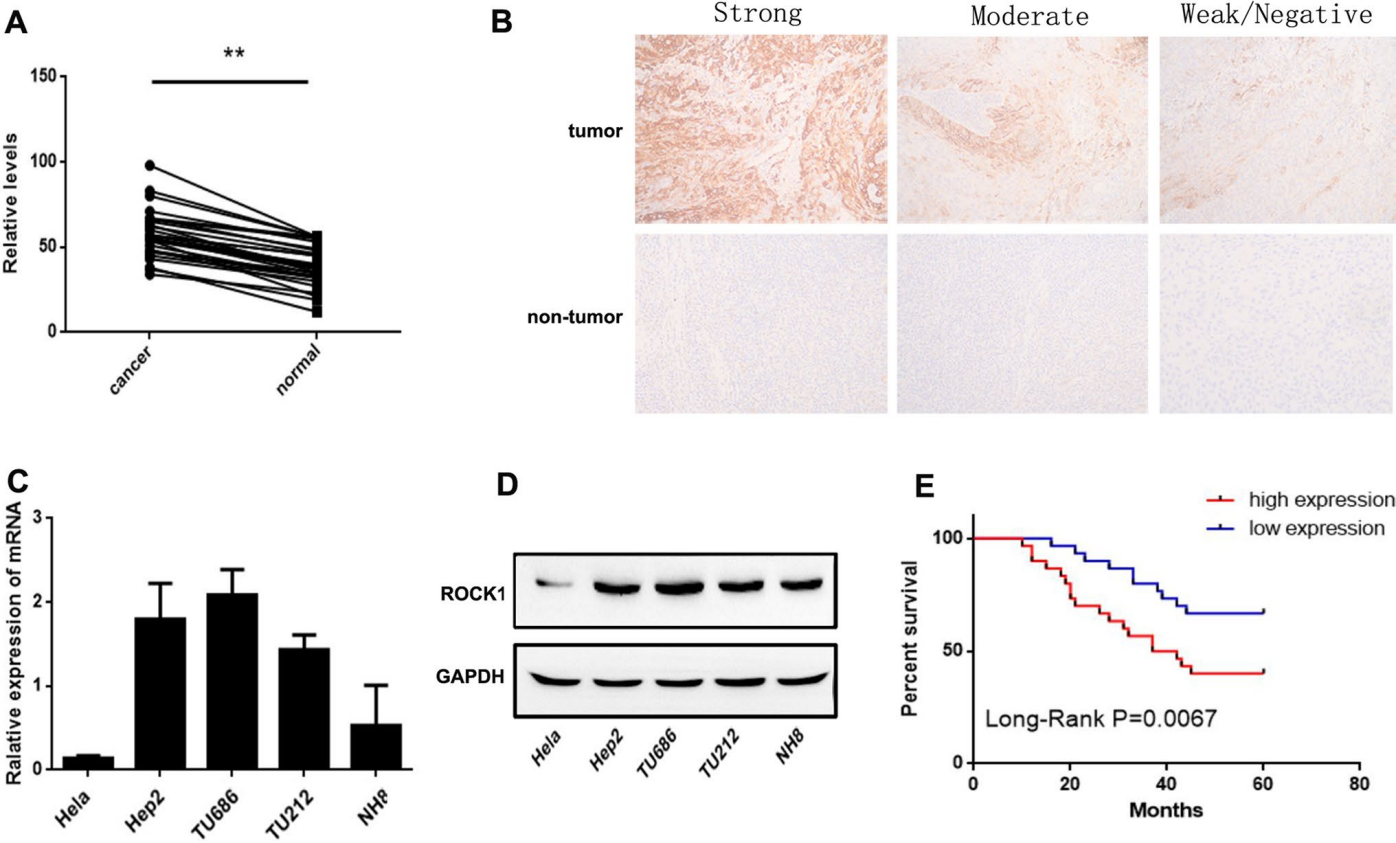
ROCK1 overexpression in human LSCC tissues and cell lines.** A** The expression levels of ROCK1 mRNA in 30 LSCC were quantified by qRT-PCR. Data are shown as 2−∆Ct (**P < 0.01). **B** IHC staining of ROCK1 in LSCC tissues (magnification: ×100). **C** The mRNA expression of ROCK1 in the LSCC cell lines was quantified by qRT-PCR. **D** The protein expression of ROCK1 in human LSCC cell lines was examined by Western Blot. **E** Patients with ROCK1 weak staining had a significantly benign prognosis than those with strong staining, P = 0.0067

To determine the role of ROCK1 in human LSCC progression, the expression level of ROCK1 was assessed in human LSCC tissues and matched adjacent non-LSCC tissues. The ROCK1 mRNA expression was examined in 30 LSCC patients by Rt-PCR. Results exhibited that it was dramatically higher in tumor tissues than matched adjacent non-tumor tissues (**P < 0.01, Fig. 1 A). In addition, the ROCK1 protein expression was examined by immunohistochemistry (IHC). 24 of 30 LSCC tissues (the positive rate: 80%) were categorized as ROCK1 protein expression positive, whereas 6 LSCC tissues and 16 adjacent normal tissues (the positive rate of adjacent normal tissues: 47%) were categorized as ROCK1 protein negative or weak expression. Moreover, the qRT-PCR and Western Blot results showed that the ROCK1 expression was higher in Hep2, NH8, TU686 and TU212 cells (Fig. 1C–D). Furthermore, the relationship between ROCK1 and clinicopathological features of 60 LSCC patients was showed in Table 1. ROCK1 with high expression in LSCC was markedly related to invasion range (P = 0.028), lymph node involvement (P = 0.019) and TNM stage (P < 0.001). But the ROCK1 expression was not related to other clinicopathological characteristics such as age (P = 0.122), tumor size (P = 0.232) or gender (P = 0.521). Furthermore, 60 LSCC patients were followed up by 5 years, which 28 patients had died. The mortality was 46.7% (28 of 60, Fig. 1E, P = 0.0067). Above observations indicate that ROCK1 is highly expressed in LSCC and related to poor survival.

**Table 1:** Relationship between ROCK1 expression level and clinicopathological characteristics in 60 LSCC patients

Clinicopathological characteristics	ROCK1 staining		P
	Weak	Strong	
Age (years)			0.122
≤ 60	14	16	
>60	11	19	
Gender			0.521
Male	20	16	
Female	18	6	
tumor size (cm)			0.232
≤ 5	19	18	
>5	20	13	
Invasion range			0.028
T1, T2	13	11	
T3, T4	20	16	
Lymph node involvement			0.019
No	9	14	
Yes	12	25	
TNM stage			0.001
I + II	8	16	
III + IV	25	11	

### ROCK1 promotes LSCC cells growth

**Fig. 2:**
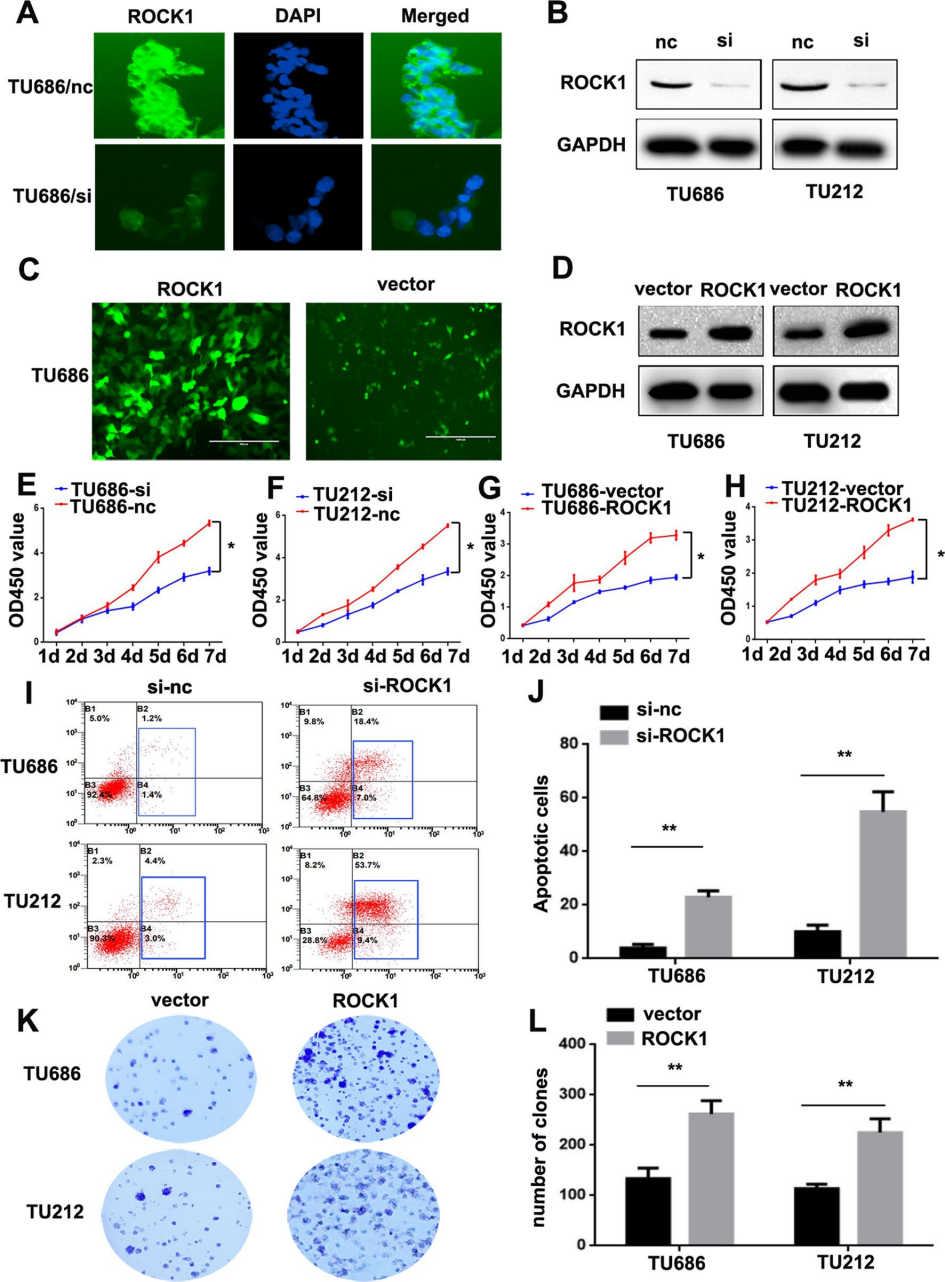
ROCK1 promotes LSCC cells growth. **A** The ROCK1 level was reduced in TU686/si-ROCK1 cells. **B** The knockdown effect was verified by Western Blot. ROCK1 was significantly decreased in TU686/si-ROCK1 and TU212/si-ROCK1 cells. **C** Plasmid transfection was used to upregulate the expression level of ROCK1 in TU686 and TU212 cells, the effect of transfection was verified via IF. **D** The expression level of ROCK1 was verified by Western Blot. ROCK1 was significantly increased in TU686/ROCK1 and TU212/ROCK1 cells. **E**,** F.** ROCK1 knockdown reduced cell proliferation (*P < 0.05). **G**, **H** Overexpression of ROCK1 increased cell proliferation (*P < 0.05). **I**,** J** ROCK1 knockdown induced cell apoptosis (**P < 0.01). **K**,** L** Overexpression of ROCK1 promoted cell proliferation (**P < 0.01)

Considering that ROCK1 is dramatically up-regulated in LSCC, it may act as an oncogene. To investigate the function of ROCK1 in LSCC cell lines, we regulate the expression level of ROCK1 in TU686 and TU212 cells. The knockdown and overexpression of ROCK1 were examined by the Immunofluorescent (IF) and Western Blot. Results showed that the ROCK1 expression was obviously cut down in TU686/si-ROCK1 and TU212/si-ROCK1 cells in comparison with negative controls (TU686/si-nc and TU212/si-nc cells, Fig. 2A–B). The ROCK1 expression was markedly enhanced in TU686/ROCK1 and TU212/ROCK1 cells in comparison with control groups (TU686/vector and TU212/vector cells, Fig. 2C–D). Distinctly, proliferation of both the TU686/si-ROCK1 and TU212/si-ROCK1 cells was lower than in negative groups (the TU686/si-nc and TU212/si-nc cells, *P < 0.05, Fig. 2E–F). Conversely, proliferation of both the TU686/ROCK1 and TU212/ROCK1 cells was higher than negative groups (the TU686/si-nc and TU212/si-nc cells, **P < 0.05, Fig. 2G–H). Moreover, the flow cytometry results showed that the percentage of apoptotic cells was significantly higher in the TU686/si-ROCK1 (22 ± 2.45) and TU212/si-ROCK1 cells (54 ± 7.42) than in negative groups (the TU686/si-nc (4 ± 1.41) and TU212/si-nc cells (10 ± 2.44), **P < 0.01, Fig. 2I–J). A colony formation assay was used to further verify the proliferation and the results exhibited the numbers of colonies in the TU686/ROCK1 and TU212/ROCK1 cells were more than the TU686/vector and TU212/vector cells (**P < 0.01, Fig. 2K–L). Together, above observations suggest ROCK1 promotes LSCC cells growth.

### ROCK1 promotes LSCC cells migration and invasion

**Fig. 3:**
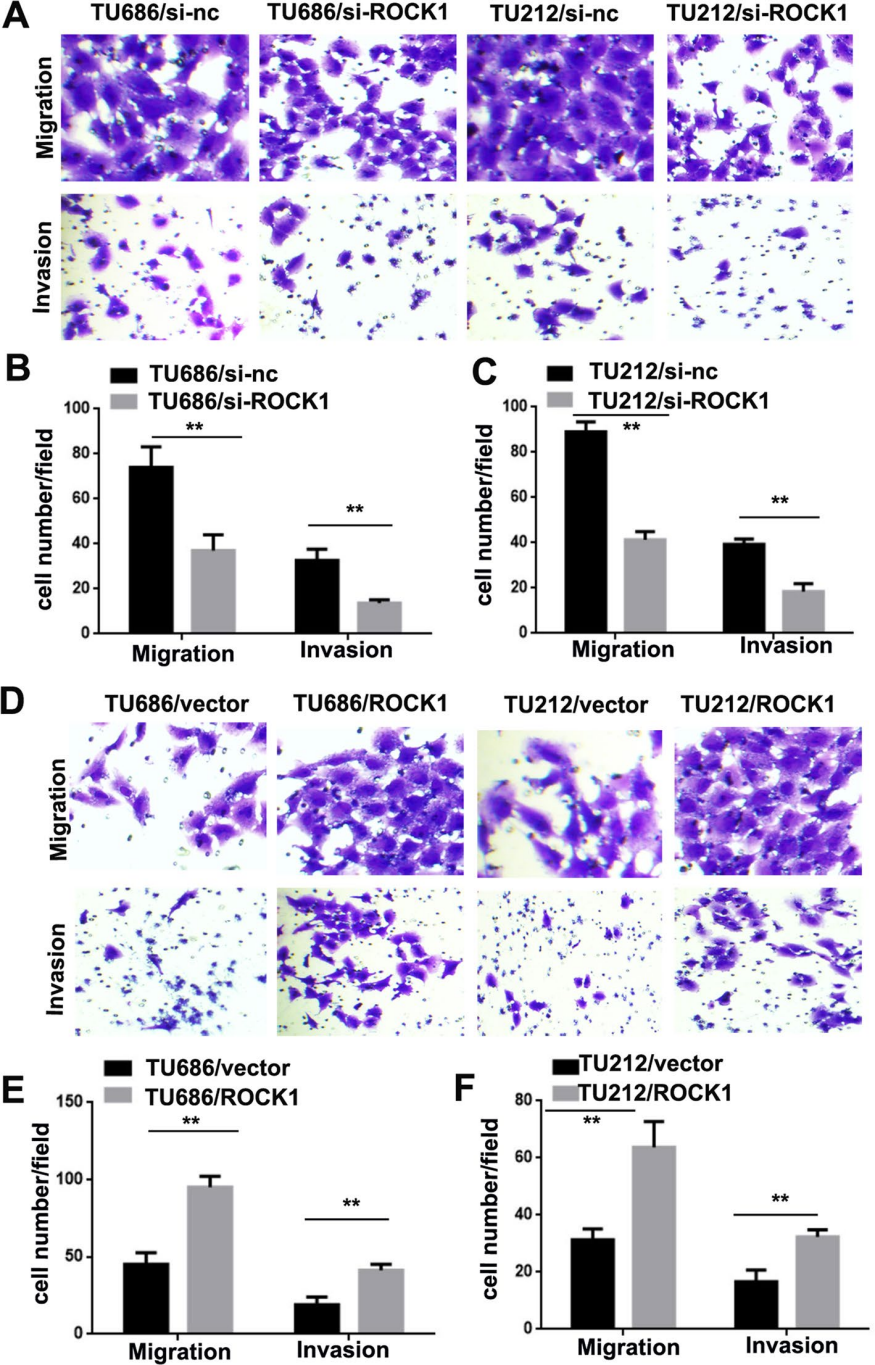
ROCK1 promotes LSCC cell migration and invasion. A–C ROCK1 knockdown reduced TU686 and TU212 cells migration and invasion (**P < 0.01). **D–F** Overexpression of ROCK1 induced TU686 and TU212 cells migration and invasion (**P < 0.01)

Transwell assays were performed to further characterize the effect of ROCK1. Results indicated more TU686/si-nc (74 ± 9.17) and TU212/si-nc cells (89 ± 4.36) migrated through Transwell chambers than TU686/si-ROCK1 (37 ± 7.0) and TU212/si-ROCK1 cells (41 ± 3.51). Finally, the invasion assay results showed that TU686/si-nc (32 ± 5.03) and TU212/si-nc cells (39 ± 2.31) invaded through Matrigel more frequently than TU686/si-ROCK1 (13 ± 1.52) and TU212/si-ROCK1 cells (18 ± 3.51, **P < 0.01, Fig. 3A–C). Conversely, less TU686/vector (45 ± 7.51) and TU212/vector cells (31 ± 3.79) migrated through Transwell chambers than TU686/ROCK1 (95 ± 7.21) and TU212/ROCK1 cells (63 ± 9.07). And the invasion assay results showed that TU686/vector (19 ± 5.00) and TU212/vector cells (17 ± 4.04) invaded through Matrigel less frequently than TU686/ROCK1 (41 ± 4.04) and TU212/ROCK1 cells (32 ± 2.52, **P < 0.01, Fig. 3D–F). Above results indicate ROCK1 promotes LSCC cells migration and invasion.

### ROCK1 may mediate FAK signaling pathway to promote the migration and invasion of LSCC

**Fig. 4:**
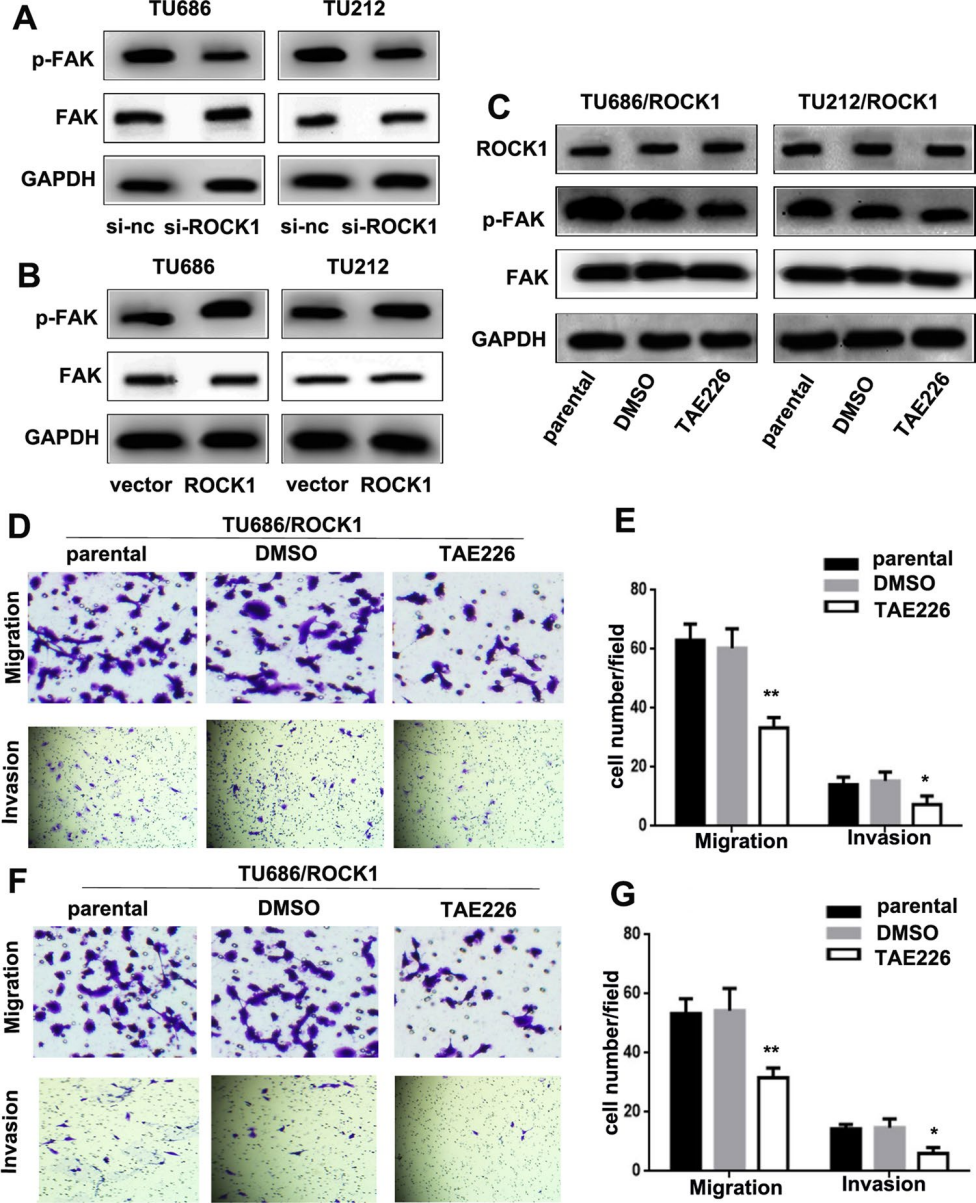
ROCK1 promotes LSCC tumorigenesis and progression via the FAK pathway.** A** The expression levels of p-FAK and FAK in TU686/si-ROCK1, TU212/si-ROCK1, TU686/si-nc and TU212/si-nc cells were examined by Western Blot. **B** The expression levels of p-FAK and FAK in TU686/ROCK1, TU212/ROCK1, TU686/vector and TU212/vector cells were examined by Western Blot. **C** The expression levels of ROCK1, p-FAK and FAK in TU686/ROCK1/parental, TU686/ROCK1/DMSO, TU212/ROCK1/parental, TU212/ROCK1/DMSO, TU686/ROCK1/TAE226 and TU212/ROCK1/TAE226 cells were examined by Western Blot. **D–G** Inhibiting FAK activity reduced TU686 and TU212 cell migration and invasion (*P < 0.05, **P < 0.01)

Accumulating evidence reveals that the FAK signaling pathway plays a crucial role in the migration and invasion of cancers [9–13]. We suggested that ROCK1 might mediate FAK signaling pathway to promote the migration and invasion of LSCC. As shown in Fig. 4 A, the expression level of p-FAK in TU686/si-ROCK1 and TU212/si-ROCK1 cells was lower than in TU686/si-nc and TU212/si-nc cells, the expression level of FAK was not altered. On the contrary, the expression levels of p-FAK in TU686/ROCK1 and TU212/ROCK1 cells were higher than TU686/vector and TU212/vector cells, the expression level of FAK was not altered (Fig. 4B). To better illustrate the effect of FAK in the tumorigenesis and progression of LSCC induced by ROCK1. A FAK inhibitor (TAE226, 2.1 μm/ml) [14], which dissolved in Dimethyl Sulfoxide (DMSO) was used to treat TU686 and TU212 cells. The expression level of p-FAK in TU686/ROCK1/TAE226 and TU212/ROCK1/TAE226 cells was lower than TU686/ROCK1/parental, TU686/ROCK1/DMSO, TU212/ROCK1/parental and TU212/ROCK1/DMSO cells, and the expressions levels of ROCK1 and FAK were not altered (Fig. 4C). Transwell assays were performed to further identify the role of FAK on the tumorigenesis and progression of LSCC cells induced by ROCK1. Results indicated more TU686/ROCK1/parental (63 ± 5.57), TU686/ROCK1/DMSO (60 ± 6.66), TU212/ROCK1/parental (53 ± 5.03) and TU212/ROCK1/DMSO cells (54 ± 7.51), migrated through Transwell chambers compared with TU686/ROCK1/TAE226 (33 ± 3.51) and TU212/ROCK1/TAE226 cells (31 ± 3.21). Finally, the invasion assays results showed that TU686/ROCK1/parental (14 ± 2.65), TU686/ROCK1/DMSO (15 ± 3.06), TU212/ROCK1/parental (14 ± 1.53) and TU212/ROCK1/DMSO cells (15 ± 3.06) invaded through Matrigel more frequently than TU686/ROCK1/TAE226 (7 ± 2.89) and TU212/ROCK1/TAE226 cells (6 ± 2.0, **P < 0.01, Fig. 4D–G). Those results suggest that the FAK signaling pathway may be involved in tumor-promotion effects induced by ROCK1 of LSCC.

### ROCK1 knockdown expression impairs LSCC metastasis in nude mice

**Fig. 5:**
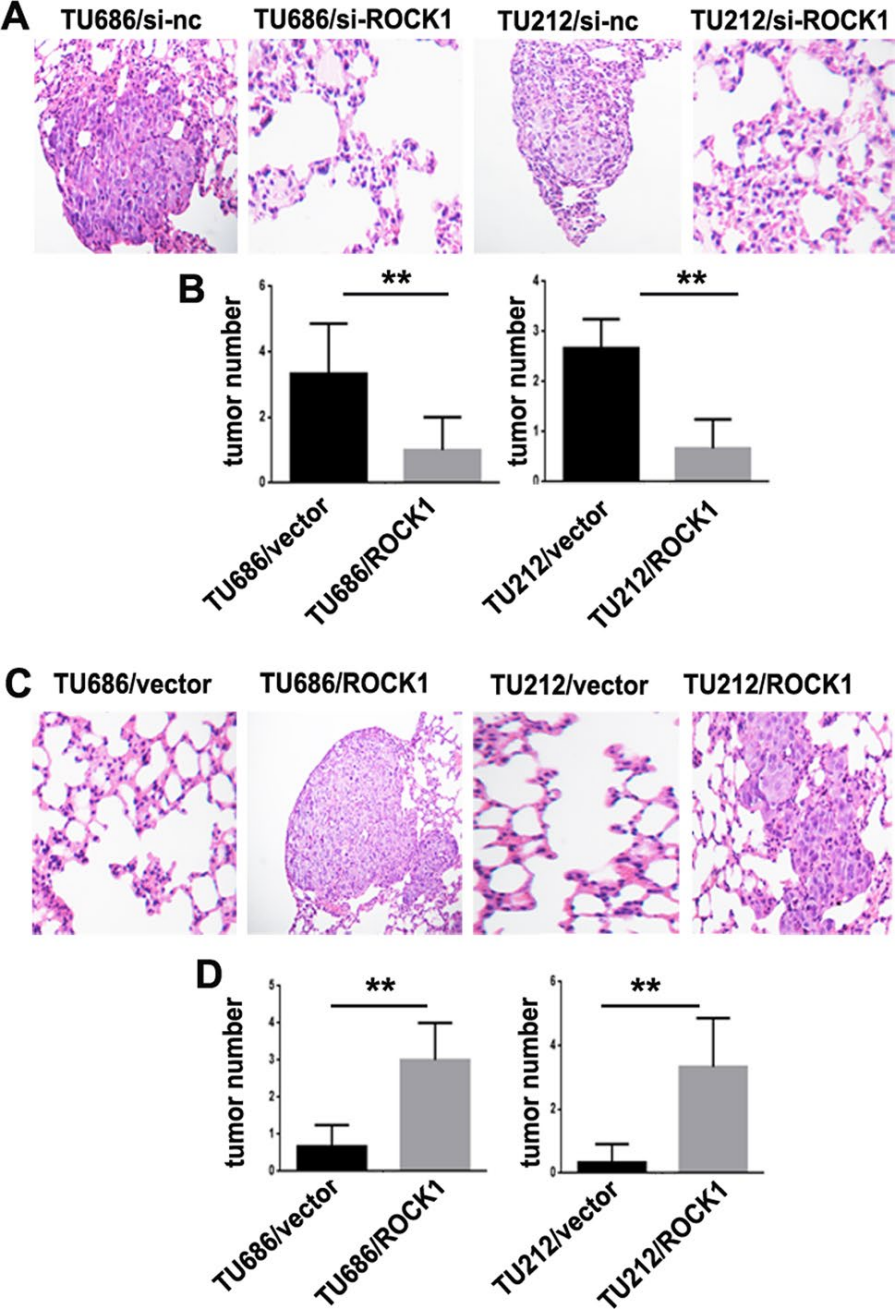
ROCK1 induces LSCC metastasis in nude mice. **A** TU686/si-ROCK1, TU212/si-ROCK1, TU686/si-nc and TU212/si-nc cells were inoculated into nude mice and pulmonary nodules were observed after 42 days (N = 5/group). H&E stains of pulmonary nodules (100×). **B** Pulmonary tissue and nodules were quantified by H&E staining from TU686/si-ROCK1, TU212/si-ROCK1, TU686/si-nc and TU212/si-nc cells (**P < 0.01). **C** TU686/ROCK1, TU212/ROCK1, TU686/vector and TU212/vector cells were inoculated into nude mice and pulmonary nodules were observed after 42 days (N = 5/group). H&E stains of pulmonary nodules (100×). **D** Pulmonary tissue and nodules were quantified by H&E staining from TU686/ROCK1, TU212/ROCK1, TU686/vector and TU212/vector cells (**P < 0.01)

In order to probe the role of ROCK1 in the tumorigenesis and progression of LSCC, TU686/si-ROCK1, TU212/si-ROCK1, TU686/si-nc, TU212/si-nc, TU686/ROCK1, TU212/ROCK1, TU686/vector and TU212/vector cells were injected into nude mice by tail vein. Six weeks after injection, TU686/si-nc (3 ± 1.00) and TU212/si-nc (3.3 ± 1.53) cells demonstrated more frequent lung metastases as compared to the TU686/si-ROCK1 (0.7 ± 0.58) and TU212/si-ROCK1 cells (0.3 ± 0.58) with lower ROCK1 expression (**P < 0.01, Fig. 5A, B). TU686/ROCK1 (3.3 ± 1.53) and TU212/ROCK1 cells (2.7 ± 0.58) demonstrated more frequent lung metastases as compared to the TU686/vector (1.0 ± 1.0) and TU212/vector cells (0.7 ± 0.58, **P < 0.01, Fig. 5C, D).

## Discussion

Dysfunctional ROCK1 leads to genetic instability, and potentially contributes to various malignant epithelial tumors which included non-small-cell lung, prostate, pancreatic, colon, esophageal cacers. Higher ROCK1 expression in NSCLC has worse survival [15]. Prostate cancer with high ROCK1 expression was markedly related to advance tumor stage, high classical and quantitative Gleason grade, positive nodal stage, positive surgical margin, and high preoperative PSA level [16]. ROCK1 activates phosphorylation at T558 to promote the breast cancer metastasis [17]. ROCK1 stimulates the growth and metastasis of pancreatic cancer (PC) cells and ROCK1 may therefore represent potential targets for clinical PC treatment [18]. Here, in our study ROCK1 is highly expressed in human LSCC tissues and cells, and high expression of ROCK1 relates with invasion range, lymph node involvement, TNM stage and poor survival prognosis for LSCC patients.

Accumulating evidence indicates that ROCK1 has an essential role in cell migration, invasion, ECM synthesis, stress-fiber assembly, mesenchymal-epithelial transition (EMT) and resistances to chemotherapy drug therapy [19–21]. Consistent with previous studies, overexpression of ROCK1 promotes the mobility, proliferation, migration and invasion of LSCC. And ROCK1 knockdown inhibits the mobility, proliferation, growth, invasion and migration in vitro, as well as pulmonary metastasis in vivo. Thus, ROCK1 promotes LSCC tumorigenesis and progression.

In our study, we further intend to investigate the molecular mechanisms of ROCK1 promotes the tumorigenesis and progression of LSCC. Numerous signaling pathways, including ERK1/2 [22], MAPK [23], VEGF [24], WNT [25] and FAK [11] signaling pathways have been reported to promote tumor tumorigenesis and progression. FAK signaling pathway is one of the most crucial ones [26]. In our present study, we find ROCK1 promotes LSCC tumorigenesis and progression via the FAK signaling pathway. ROCK1 knockdown reduces the expression level of p-FAK and consequently decreases the proliferation, invasion and migration of LSCC. On the contrary, overexpression of ROCK1 increases the p-FAK and promotes the proliferation, invasion and migration of LSCC. Furthermore, TAE226, a FAK inhibitor, is applied to explore its role in promoting LSCC tumorigenesis and progression. All results shows that ROCK1 mediates FAK to promote LSCC tumorigenesis and progression. And ROCK1 may therefore represent potential targets for clinical LSCC treatment.

Nonetheless, more experiments should be conveyed the connection between ROCK1 and FAK signaling pathway rather than other signaling pathways mentioned above. In the study, we had inhibited FAK with the treatment of its inhibitor (TAE226). And the result indicated that inhibiting FAK activity impaired LSCC cellular migration and invasion. For in vivo study, we did not measure the expression of p-FAK and FAK, because we think the differences with drug treatment could be due to systemic effects, not necessarily effects on tumor cells, or the process of metastasis itself. Therefore, in our further study, we will knock-down FAK in vivo to explore the effect of FAK in LSCC metastasis. Moreover, further preclinical trials and relevant researches should be carried out to examine the effect and safe of drugs.

To sum up, we demonstrate that ROCK1 promotes LSCC tumorigenesis and progression via activation of the FAK signaling pathway. Targeting the ROCK1 molecule might serve as potential targets for LSCC clinical treatment.

## Data Availability

The data and material during the current study were available from the corresponding author on reasonable request.
